# Males, the Wrongly Neglected Partners of the Biologically Unprecedented Male–Female Interaction of Schistosomes

**DOI:** 10.3389/fgene.2019.00796

**Published:** 2019-09-06

**Authors:** Zhigang Lu, Sebastian Spänig, Oliver Weth, Christoph G. Grevelding

**Affiliations:** ^1^Wellcome Sanger Institute, Wellcome Genome Campus, Hinxton, United Kingdom; ^2^Insitute for Parasitology, BFS, Justus Liebig University Giessen, Giessen, Germany; ^3^Department of Mathematics & Computer Science, University of Marburg, Marburg, Germany

**Keywords:** schistosomes, male–female interaction, transcriptomics, pairing-dependent gene expression, TGFβ signaling, neuropeptide, G protein-coupled receptor

## Abstract

Schistosomes are the only platyhelminths that have evolved separate sexes, and they exhibit a unique reproductive biology because the female’s sexual maturation depends on a constant pairing contact with the male. In the female, pairing leads to gonad differentiation, which is associated with substantial morphological changes, and controls among others the expression of gonad-associated genes. In the male, no morphological changes have been observed after pairing, although first data indicated an effect of pairing on gene transcription. Comprehensive transcriptomic approaches have revealed an unexpected high number of genes that are differentially transcribed in the male after pairing. Their identities suggest roles for the male that are not restricted to feeding and enhanced muscular power to transport paired female and, as assumed before, to induce its sexual maturation by one “magic” factor. Instead, a more complex picture emerges in which both partners live in a reciprocal sender-recipient relationship that not only affects the gonads of both genders but may also involve tactile stimuli, transforming growth factor β signaling, nutritional parts, and neuronal processes, including neuropeptides and G protein-coupled receptor signaling. This review provides a summary of transcriptomics including an overview of genes expressed in a pairing-dependent manner in schistosome males. This may stimulate further research in understanding the role of the male as the recipient of the female’s signals upon pairing, the male’s “capacitation,” and its subsequent competence as a sender of information. The latter process finally transforms a sexually immature, autonomous female without completely developed gonads into a sexually mature, partially non-autonomous female with fully differentiated gonads and enormous egg production capacity.

## Schistosomes and the Male–Female Interaction

Schistosomes are parasitic platyhelminths causing schistosomiasis (bilharzia), an infectious disease of worldwide importance for humans and animals. The World Health Organization has listed schistosomiasis as one of the neglected tropical diseases. More than 200 million people required preventive treatment in 2016 (World Health Organization, 2019; McManus et al., 2018). This disease is most prevalent in Africa but occurs also in Asia and South America due to the presence of tropical water snails as intermediate hosts, which prefer warm (sub)tropical habitats. Unexpectedly, there is recent evidence of an autochthonous site also in southern Europe (Corsica), where the snail-host species occurs due to moderate climate conditions (Boissier et al., 2016).

As a waterborne disease, schistosomiasis affects humans and animals exposed to water infested with cercariae, the infectious larval stage originating from snails. Some schistosome species comprise zoonotic potential, which increases the risk of infection (Standley et al., 2012). Together, these facts make disease control difficult and contribute to an additional, socioeconomic problem (Garchitorena et al., 2017).

The pathology of schistosomiasis is triggered by eggs that paired females deposit in the bloodstream of vertebrate hosts. These eggs eventually lodge in organs such as the liver where they cause inflammation and fibrosis (Olveda et al., 2014; McManus et al., 2018). The prerequisite for egg production is the complete development of the female gonads. This, however, is only achieved if a constant pairing contact with a male has been established. To this end, the female resides within a ventral groove formed by the male, the gynecophoral canal. This close liaison can last over years, an exceptional phenomenon in nature (Basch, 1991; Grevelding, 2004). Males play a pivotal role in controlling schistosome reproduction by inducing mitoses and differentiation processes in the reproductive organs (ovary and vitellarium, the latter providing vitelline cells for the production of mature eggs) of the paired female, a process that comes along with a significant increase of its body size (Popiel and Basch, 1984a; Den Hollander and Erasmus, 1985; Kunz, 2001). Pairing even controls the expression of female-specific expressed genes with functions in the vitellarium (LoVerde and Chen, 1991; Grevelding et al., 1997). Although the molecular consequences of pairing on females have been a strong focus of basic research (Hoffmann, 2004; LoVerde et al., 2009; Beckmann et al., 2010), pairing-dependent processes in males are somewhat neglected.

## A Historical Snapshot of the Male Schistosomes’ Perspective

With respect to their sexual biology, schistosome males appear “ready to go.” Being independent of pairing, they possess fully developed testes and seminal vesicles filled with differentiated sperms as confirmed by morphological analyses (Neves et al., 2005; Beckmann et al., 2010). Sperm was excluded as a factor inducing the sexual maturation of the female, and irradiated or surgically manipulated males lacking testes were still capable of mating and inducing sexual maturation in paired females as well as egg production (Armstrong, 1965; Michaels, 1969). Alternatively, the male was proposed to deliver specific molecules during pairing that supervise body length and the sexual maturation of the female (Armstrong, 1965; Basch and Basch, 1984), which depends on the pairing status; unpaired females are infertile because their reproductive organs have not fully developed (Neves et al., 2005; Beckmann et al., 2010). Furthermore, female sexual maturation was hypothesized to be a consequence of local activities of molecules (Michaels, 1969; Popiel and Basch, 1984b). In addition, a tactile impulse was proposed (Basch and Basch, 1984). Finally, a male-secreted hormonal factor(s) was suggested to be transferred to the female (Ruppel and Cioli, 1977; Shaw et al., 1977; Atkinson and Atkinson, 1980; Basch and Nicolas, 1989). However, none of these hypotheses resulted in the identification of the “magic male factor”.

From the metabolic perspective, glucose and cholesterol were demonstrated to be delivered by the male during pairing, and it was hypothesized that nourishment contributes to female sexual maturation (Conrford and Huot, 1981; Cornford and Fitzpatrick, 1985; Haseeb et al., 1985; Silveira et al., 1986). The most persuasive evidence for an important player in the game resulted from studies about the gynecophoral canal protein (GCP). First detected in adult *Schistosoma mansoni*, *Sm*GCP was identified as a glycoprotein putatively transferred from the male to the female (Gupta and Basch, 1987) and later, by immunolocalization, to be widely distributed on the surface of a paired female (Bostic and Strand, 1996). Structurally, *Sm*GCP lacks a transmembrane domain but reveals short, conserved repeat regions with sequence similarity to fasciclin I, a neuronal cell-adhesion protein. In males, *Sm*GCP expression appeared to be limited to the gynecophoral canal region, the mating partners’ interface. Furthermore, *Sm*GCP seemed to be down-regulated in unpaired males. These findings suggested that *Sm*GCP is diffusible and delivered by the male during a pairing contact (Bostic and Strand, 1996). Indeed, results of a subsequent study in *Schistosoma japonicum* indicated the importance of *Sj*GCP for pairing. RNA interference experiments against *Sj*GCP resulted in reduced pairing stability *in vitro* and *in vivo* (Cheng et al., 2009). Finally, evidence was found for the regulation of *Sm*GCP *via* transforming growth factor β (TGFβ)-dependent signaling in *S. mansoni* (Osman et al., 2006). Although the biochemical activity of GCP has not yet been clearly addressed, there is accumulating evidence for its participation in male–female interaction.

In earlier studies, the DNA synthesis marker [^3^H]thymidine was used in incorporation assays with females *in vitro* to determine the mitosis rates dependent on the pairing. Comparing females paired in the presence of thymidine to either pairing-experienced males (bM, bisex males) or pairing-inexperienced males (sM, single-sex males) demonstrated that maturity is decisive. To induce mitogenic activity in females, sM required a significantly longer mating period (≥24 h) than bM (Den Hollander and Erasmus, 1985), which stimulated mitogenic activity in females within the first 24 h of pairing. This early study already pointed toward bidirectional communication between the partners during the initial phase of pairing. Furthermore, this result suggests that males have to pass through a process of capacitation before they acquire competence to supervise female sexual maturation — part of which is the induction of mitoses (Knobloch et al., 2002).

## Transcriptomic Performance of Male Schistosomes

During the last 15 years, *omic* studies have allowed unprecedented insights in the life processes of a great variety of organisms (Weissenbach, 2016), including schistosomes (Verjovski-Almeida et al., 2003; Hu et al., 2003; Berriman et al., 2009; Schistosoma japonicum Genome Sequencing and Functional Analysis Consortium, 2009; Protasio et al., 2012; Young et al., 2012; Anderson et al., 2015; Smit et al., 2015; Cai et al., 2016; Sotillo et al., 2017; Wang et al., 2017; Giera et al., 2018). Whereas the majority of these studies applied RNA-seq techniques, microarray analyses and serial analysis of gene expression (SAGE/SuperSAGE) were alternatively used. Among others, these techniques were also applied to compare bM and sM. One SAGE-based approach found differential regulation for transcripts contributing to developmental processes, metabolism, and the redox system (Williams et al., 2007). Even before the genome project was finished, an early microarray analysis found 30 genes to be exclusively transcribed in bM and 66 in sM (Fitzpatrick and Hoffmann, 2006). The identities of these differentially expressed genes indicated their involvement in RNA metabolic processes, which was independently supported in another microarray study (Waisberg et al., 2007). In another approach combining SuperSAGE (a second-generation SAGE technique allowing the identification of longer RNA sequence tags) and microarray analyses, corresponding data sets were produced to get a comprehensive overview of genes differentially transcribed between bM and sM. Among 6326 sense transcripts detected by both analyses, 29 were found to be significantly differentially transcribed (Leutner et al., 2013). Besides differences in the transcript levels of genes involved in metabolic processes, evidence was obtained for additional differences in neuronal processes and TGFβ signaling. In this context, a *S. mansoni* ortholog of follistatin (*Sm*Fst; Smp_123300) was found to be differentially transcribed with an interesting bias toward sM. The latter was independently confirmed by a subsequent RNA-seq approach including paired and unpaired adults and their gonads (Lu et al., 2016; Lu et al., 2017) as well as by independent quantitative polymerase chain reaction (qPCR) analyses (Leutner et al., 2013; Haeberlein et al., 2019). Based on the corresponding results from microarray, SuperSAGE, RNA-seq, and qPCRs, all exhibiting higher transcript occurrence in sM, *Sm*Fst is probably the most intensively studied gene with respect to expression profiling. Thus, it can be used in future studies as a marker for differential transcription in bM versus sM (Haeberlein et al., 2019). Follistatins are known antagonists in TGFβ signaling pathways and block ligands of the TGFβ family such as TGFβ, activin, and bone morphogenetic protein (BMP) (Massagué and Chen, 2000; Moustakas and Heldin, 2009; Heldin and Moustakas, 2016). The first characterization of *Sm*Fst showed testes localization (by *in situ* hybridization). By yeast two-hybrid analyses, an interaction potential with *S. mansoni* orthologs of the TGFβ ligands *Sm*InAct and *Sm*BMP was found. The agonists colocalized with *Sm*Fst in the testes (Leutner et al., 2013). These results suggest that TGFβ signaling also plays a role in male-female interaction and is part of the bidirectional communication between both genders. As such, *Sm*Fst could represent one of several competence factors of males expressed in response to pairing. Indeed, a recent *in vitro* study with paired, separated, and re-paired males demonstrated an immediate influence of pairing on the on/off transcriptional status of *Sm*Fst (Haeberlein et al., 2019). This finding adds to previous hypotheses that TGFβ signaling is involved in pairing-dependent reproductive processes in schistosomes (LoVerde et al., 2007; Buro et al., 2013). One role of *Sm*Fst in sM might be the prevention of the activation of one or more of its TGFβ pathways before pairing. Figuratively seen, *Sm*Fst in sM appears like a systemic handbrake of a specific, male competence-related biological driving route needed after pairing to reach maturation – a hypothesis that awaits corroboration.

Today, RNA-seq represents the state-of-the-art technology for transcriptome analysis providing both a wide analytical range and the quantification of study samples. Theoretically, RNA-seq can cover all transcripts of a biological sample, an advantage over microarrays or SAGE/SuperSAGE (Marioni et al., 2008). Recently, RNA-seq was applied for comparative transcript profiling in paired and unpaired adult *S. mansoni* and their gonads. Of more than 7,000 transcripts detected in the gonads, 243 (testes) and 3,600 (ovaries) were transcribed in a pairing-dependent manner. In addition to genes preferentially or specifically transcribed in adults and gonads of both genders, evidence was obtained for pairing-dependent processes in the gonads affecting genes with, for example, stem cell-associated functions. This was particularly expected for females due to the pairing-induced differentiation processes in the vitellarium and the ovary (Erasmus, 1973; Shaw, 1987; Kunz, 2001; Neves et al., 2005; Beckmann et al., 2010). Remarkably, from their annotation, many differentially transcribed genes appeared to be involved in neuronal processes. This perception substantiated one of the results of the combinatory SuperSAGE/microarray approach comparing sM and bM transcript profiles (Leutner et al., 2013).

## “Nervous” Male Schistosome

**Figure 1:**
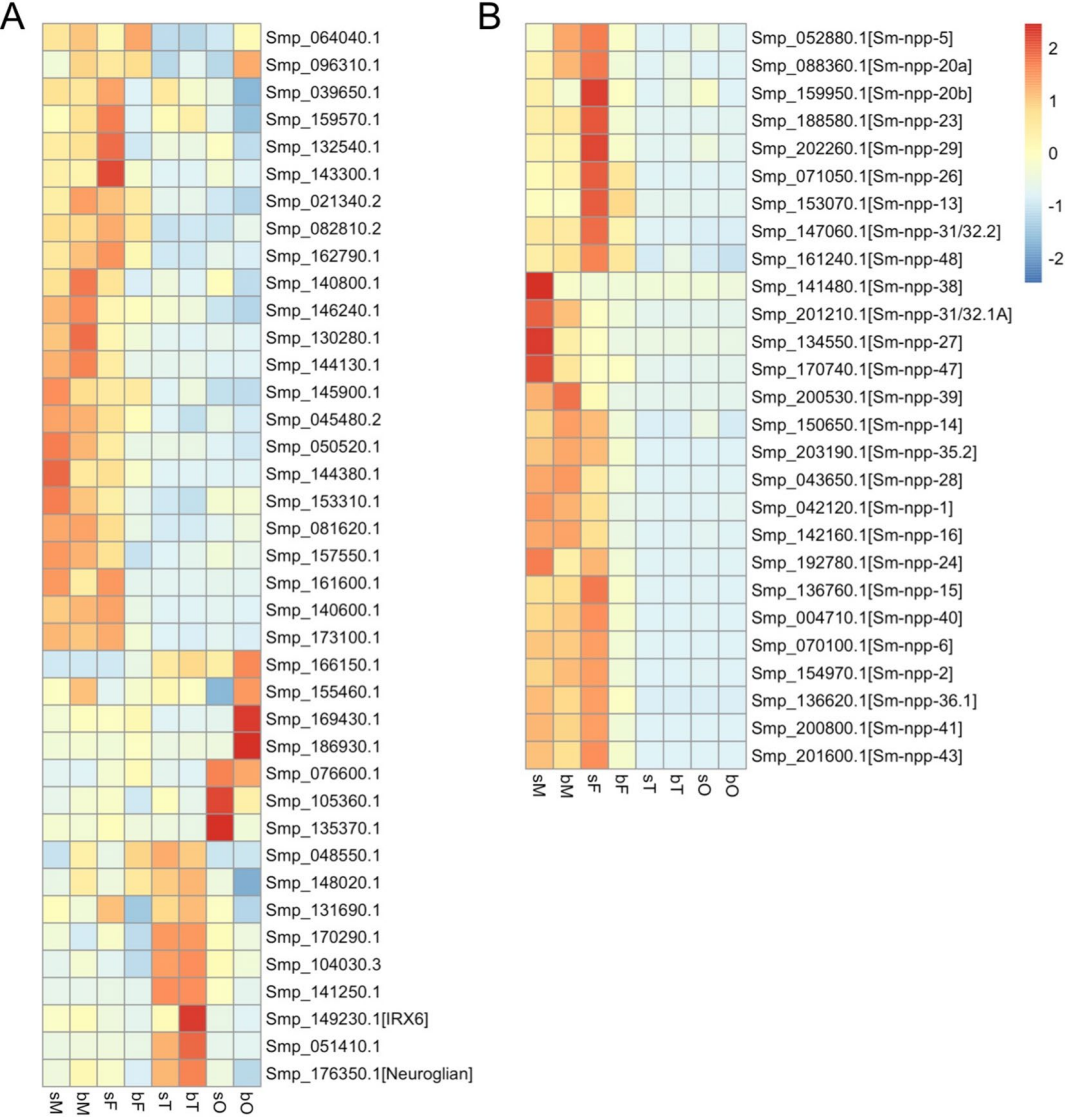
Heatmaps showing the relative expression of genes annotated as neural genes **(A)** (Berriman et al., 2009, Supplementary Table 9: (https://media.nature.com/original/nature-assets/nature/journal/v460/n7253/extref/nature08160-s2.xls)) and genes potentially coding for neuropeptide precursors **(B)** (Koziol et al., 2016, **Supplementary Data S3**: (https://www.sciencedirect.com/science/article/abs/pii/S0020751916301217?via%3Dihub)) in *S. mansoni* adults and gonads (red, up-regulation; blue, down-regulation). sM, unpaired males; bM, paired males; sF, unpaired females; bF, paired females; sT, testes of sM; bT, testes of bM; sO, ovaries of sF; bO, ovaries of bF.

One objective of human neuroscience is to understand how neuronal circuits direct behavior, how humans perceive the world, how they learn from experience, how memory works, how movements are directed, and how communication is realized (National Research Council (US) Committee on Research Opportunities in Biology, 1989). The basis for integrating all these interactions and requirements *via* neuronal circuits was laid in evolution. In principle, similar objectives also apply to schistosomes. From the male’s perspective, the questions are (i) how does it behave within the final host perceiving its environment to organize migration to target locations such as the portal system of the liver, (ii) how does it find its mating partner, (iii) how does it “learn” from a first pairing experience (capacitation and gaining competence), (iv) how does it move from the liver further on to the mesenteric veins in the gut area while carrying its mate inside the gynecophoral canal, and (v) how does the male “communicate” with its partner during this process and later on after reaching the final destination to organize large-scale egg production and longevity? It appears obvious that molecular communication at different parallel levels is part of the answer, and all transcriptomics data obtained thus far are in favor of this assumption. Data analyses have indicated among others that regulatory RNAs (Cai et al., 2016; Zhu et al., 2016) and kinase activity (Grevelding et al., 2018) but also neuronal regulation are involved (Cai et al., 2016; Wang et al., 2017; Hahnel et al., 2018). In an RNA-seq analysis of *S. mansoni* (Lu et al., 2016; Lu et al., 2017), 39 genes with potential function in neuronal processes (Berriman et al., 2009) were identified to be transcribed in the adult stage with varying transcript levels in whole worms as well as gonads and, to a large extend, in a pairing-dependent manner (**Figure 1A**). Many of these genes were found to be preferentially transcribed in bM and sM but also in unpaired females (sF, single-sex females) (Lu et al., 2016). Remarkably, the transcript levels of 64% of these genes decreased in mature females (bF, bisex females) after pairing. Similarly, transcripts of some genes detected in ovaries (bO, ovaries of bF; sO, ovaries of sF) and testes (bT, testes of bM; sT, testes of sM) occurred in a tissue-preferential and/or pairing-dependent manner. This included genes with functions in neuronal stem cells such as (i) an ortholog of IRX6 (Smp_149230), which is a homeodomain transcription factor of the iroquois family known to regulate interneuron development (Star et al., 2012) as well as germ-cell maturation in gonads (Kim et al., 2011), and (ii) a neuroglian ortholog (Smp_176350), possibly involved in regulating neuronal circuits (Boerner and Godenschwege, 2010). Of these, a neuroglian ortholog was also listed as a gene showing male-biased transcript occurrence in *S. japonicum* (Cai et al., 2016).

Additional support was obtained from studies about neuropeptidergic signaling, which was discussed playing fundamental roles in flatworm locomotion, feeding, host finding, regeneration, and reproduction (McVeigh et al., 2005; McVeigh et al., 2009; Collins et al., 2010). In an *in silico* analysis, 46 potential flatworm neuropeptides emerging from 32 neuropeptide precursors (npps) have been predicted (Koziol et al., 2016). Of these, transcripts of 7 *npp* genes were localized in the protoscolex of *Echinococcus multilocularis* and appeared to be expressed in the nervous system. In another study, RNA-seq data of the schistosome esophagus area showed that transcripts of one of the *npps* (*Sm_npp*_20a; Smp_088360 with transcripts enriched in males and unpaired females but not in gonads; **Figure 1B**) are enriched in the head part of male worms (Wilson et al., 2015). Furthermore, transcriptional profiling comparing *S. mansoni* female head and tail showed that 23 of 27 listed *Sm_npps* expressed in adult *S. mansoni* (**Figure 1B**) preferentially localized in the head part (Wang and Collins, 2016). Therefore, we assume that these NPPs are enriched in the head, possibly in the nervous system. Transcript levels of almost all 27 *Sm_npps* dominated in bM, sM, and sF (**Figure 1B**). Although transcript levels may not be representative for protein levels and/or protein half time, and although also a low amount of protein can be of high cell biological and/or physiological importance, it is tempting to speculate that the comparatively reduced *Sm_npp* transcript levels in bF may point to a lower importance of these neuropeptides (and associated neuronal processes) for females after pairing. In contrast, females before pairing may have different physiological requirements comprising more neuronal and further processes. This view is in line with previous studies concluding that, from a transcriptomic point of view, schistosome females express divergent gene repertoires regulated by pairing (Fitzpatrick and Hoffmann, 2006) and unpaired females are much closer related to males than to paired females (Lu et al., 2016; Grevelding et al., 2018). In view of the male–female interaction of *S. mansoni*, these results also suggest that neuropeptide-mediated regulation circuits are more active in males than in paired females, which may point to a higher importance of neuronal processes for males. Interestingly, a similar tendency with respect to *npp* gene transcription was found in a study about male and female *S. japonicum* (Wang et al., 2017). Among others, the authors investigated different time points throughout the sexual developmental, from pairing to maturation. This included day 16 after final-host infection, when pairing starts, to day 28, when paired females produce eggs. Based on a search for *npp* orthologs of *S. japonicum* in WormBase Parasite (https://parasite.wormbase.org) and looking for their transcript profiles within the data set provided by Wang et al. (2017), a clear tendency can be registered for a reduction of *Sj_npp* transcript levels in females after pairing from day 18 on. In contrast, the transcript levels of these *Sj_npps* remained at a constant level or, in one case (Sjp_0097680, potential ortholog of Smp_052880), increasing level from day 18 on (**Supplementary Data 1**). Figuratively, one could think that schistosome females “hand over” the responsibility for maintaining most of the neuronal circuits involving neuropeptide signaling to their partners after establishing the pairing contact. Indirect support for this assumption was obtained by the analysis of *S. mansoni* G protein-coupled receptors (GPCRs), of which some might represent *Sm_npp* targets. Based on the RNA-seq data, a comparative analysis of the GPCR*ome* generally revealed a pattern of transcriptional activity for the majority of the investigated GPCRs that resembled the patterns of the majority of *Sm_npps*: compared to bF, transcripts of these GPCRs occurred in a higher abundance in sM, bM, and Sf (Hahnel et al., 2018). Preliminary data of a deorphanization approach to uncover GPCR-neuropeptide interaction are in support of the assumption that some of the *Sm-npps* and GPCRs correspondingly regulated in the mentioned way may indeed interact (Weth et al., *in preparation*).

Finally, a detailed analysis of transcript levels of two additional genes involved in neuronal processes fits into this scenario. These genes are *S. mansoni* orthologs of a dopa decarboxylase/tyrosine decarboxylases (*Sm-tdc*, Smp_135230), involved in neurotransmitter metabolism (De Luca et al., 2003), and *ebony* (*Sm-ebony*, Smp_158480), a gene controlling neurotransmitter inactivation (Richardt et al., 2003; Hartwig et al., 2014). Transcript profiling by RNA-seq showed high transcript levels of *Sm-tdc* in bM compared to sM and no transcripts in bF or sF. The meta-analysis of all life stages showed that *Sm-tdc* is stage- and gender-specific expressed in males, being significantly up-regulated after pairing (Lu and Berriman, 2018). Similarly, the amount of *Sm-ebony* transcripts dominated in bM but also in sF, whereas sM and bF showed significantly reduced transcript levels. The meta-analysis showed stage-specific expression in adults with a significant up-regulation in bM and sF (Lu and Berriman, 2018). A comprehensive quantitative reverse transcription-PCR analysis with RNA from males after pairing, separation, and re-pairing *in vitro* finally confirmed the direct influence of pairing on the transcript levels of both genes with a clear bias toward bM (Haeberlein et al., 2019). With respect to *Sm-tdc* and *Sm-ebony*, similar pairing-influenced transcript patterns were found for the orthologs, *Sj*AADC and *Sj-ebony*, of *S. japonicum*. Wang et al. (2017) observed an increase of *Sj*AADC (Sjp_0075370) and *Sj-ebony* (Sjp_0068110) transcripts in males after pairing, whereas in females transcript levels of these genes remained constant at a low level after pairing. The localization of *Sj*AADC transcripts at the gynecophoral canal region (Wang et al., 2017), the interface between paired male and female schistosomes, further supports the view that neuronal processes may govern at least part of the male-female interaction.

## Overview of Genes Differentially Transcribed in Paired Versus Unpaired Males

Based on the existence of three transcriptomics data sets for schistosome males, an overview was generated about all genes commonly found to be significantly differentially transcribed between bM and sM. These data sets were independently obtained by different methods with varying pros and cons depending on the technical basis of these methods (microarray, SuperSAGE, and RNA-seq; for details, see Leutner et al., 2013; Lu et al., 2016; Lu et al., 2017). However, these data sets were produced with RNA of the same origin, a Liberian strain of *S. mansoni* (Grevelding, 1995).

**Figure 2:**
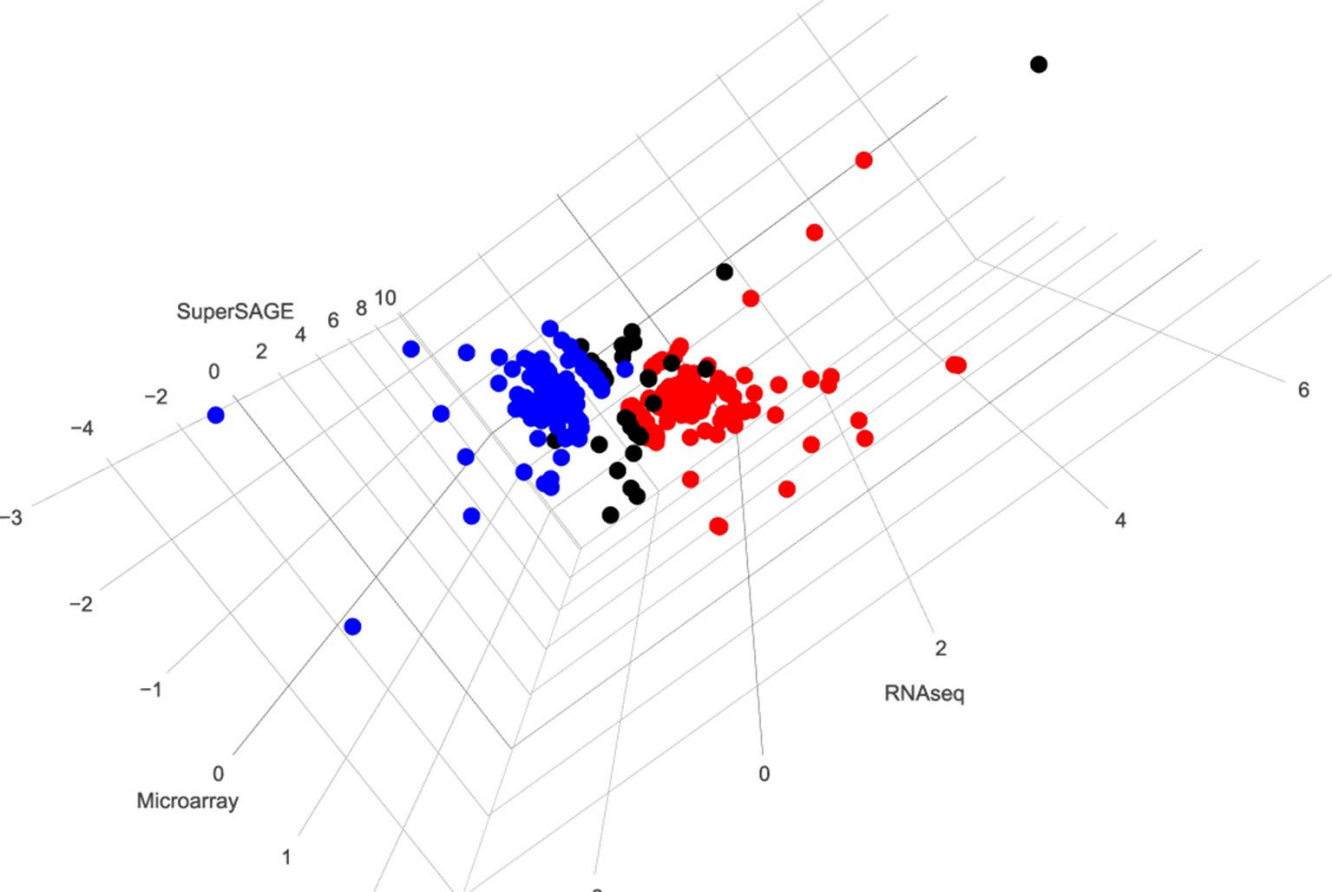
3D visualization of genes whose expression is significantly regulated by pairing in at least two data sets. Red (154) and blue (153) dots indicate genes found to be up- or down-regulated, respectively, after pairing as identified by at least two approaches. Black dots (38) indicate different regulation patterns in three data sets. To generate this overview, data from microarray, SuperSAGE analyses (Leutner et al., 2013), and RNA-seq (Lu et al., 2016) were extracted from the respective supplementary tables. To simplify the comparison process, representative gene identifiers were used for all three data sets. In case of multiple-transcript genes, the values were compared among the data sets before one representative was chosen. Significantly differentially regulated genes were selected using the same threshold used in the mentioned studies: q < 0.01 for microarray, p < 1e-10 for SuperSAGE, and FDR < 0.05 for RNA-seq. Transcripts meeting the criteria of occurrence in at least two approaches were picked, and the corresponding genes were visualized using the R package plotly (https://plot.ly) [(**Supplementary Table 1**: http://schisto.xyz/male-deg-3d/ [Interactive visualization of differentially expressed genes between bM and sM from three approaches. The users can use the mouse to zoom in/out and rotate the plot as well as to see detailed information for each gene.)].

After merging these three data sets, transcripts of 5352 genes were detected by all approaches. Applying the same significance cutoff values used before in these studies (RNA-seq FDR < 0.05; microarray q < 0.01; SuperSAGE p < 1e-10), 154 genes were found to be significantly up-regulated and 153 genes significantly down-regulated after pairing as identified by at least two approaches (**Figure 2** and **Supplementary Table 1**). In particular, we identified 43 genes that were up-regulated (21; bM > sM) or down-regulated (22; bM < sM) in all three data sets, including follistatin (highlighted in **Supplementary Table 1**; sheet 2, no. 21).

## Conclusion and Perspective

The analysis of transcriptomic data obtained thus far has provided conclusive evidence for a substantial molecular contribution of the male to the male–female interaction and the reproductive biology of schistosomes. The male’s effort is not restricted to nutritional support and muscle power for carrying the paired female around, which lodges inside the male’s gynecophoral canal. Instead, the pairing scenario appears more complex, involving different signaling systems mediating communication between the partners. This includes neuronal processes whose management asymmetrically shifts to the male side upon pairing. Therefore, this review about the males’ perspective of the reciprocal sender-recipient relationship of schistosome couples may stimulate future research in this area. Understanding male–female interaction will give a twofold return: (i) for basic science, solving one of the most interesting but yet unanswered question of schistosome biology, and (ii) and for applied research in view of the high demand finding alternative treatment concepts to fight schistosomiasis (Bergquist et al., 2017).

## Data Availability

Publicly available datasets were analyzed in this study. This data can be found here: https://www.ncbi.nlm.nih.gov/pmc/articles/PMC4976352/. 

## Author Contributions

ZL, SS, and OW prepared data and figures and substantially contributed to the work. CG conceived and wrote the manuscript.

## Funding

Studies leading to this review were supported by grants of the Wellcome Trust (FUGI, 107475/Z/15/Z) and the Deutsche Forschungsgemeinschaft (GR 1549/7-3).

## Conflict of Interest Statement

The authors declare that the research was conducted in the absence of any commercial or financial relationships that could be construed as a potential conflict of interest.

## Supplementary Material

The Supplementary Material (**Supplementary Table 1** and **Supplementary Data 1**) for this article can be found online at: https://www.frontiersin.org/articles/10.3389/fgene.2019.00796/full#supplementary-material
