# Supplementary material for: Males, the Wrongly Neglected Partners of the Biologically Unprecedented Male–Female Interaction of Schistosomes
Source: Front Genet. 2019 Sep 6;10:796. doi: 10.3389/fgene.2019.00796 (PMC6743411; doi:10.3389/fgene.2019.00796)
Supplement: Supplementary file 2 [file DataSheet_1.pdf]

Supplementary data 1

Overview of the transcript profiles of the potential *npp* genes in *S. japonicum*.

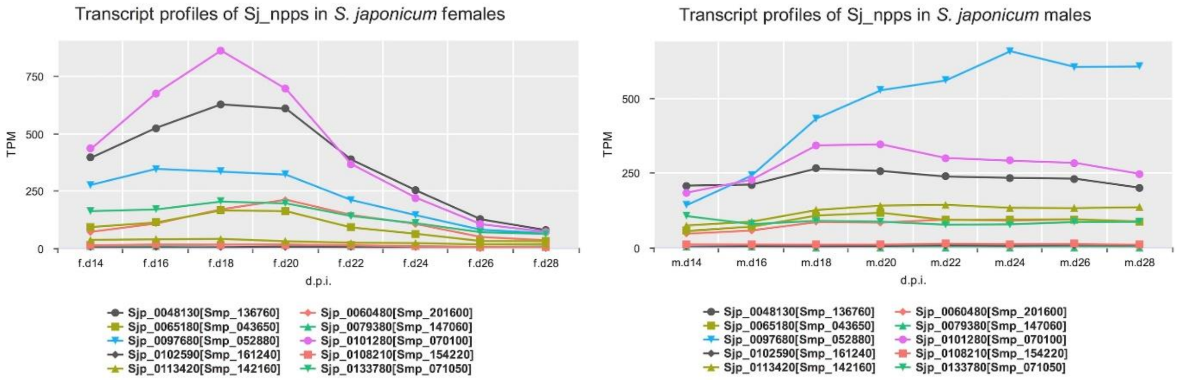

Ten *npps* of *S. japonicum* (*Sjp_* ...; each color is representative for one *Sjp_npp*) were identified by using all *npp* genes of *S. mansoni* [Smp_ …] as templates for a search of potential orthologs in *S. japonicum* using the BioMart module from WormBase ParaSite (https://parasite.wormbase.org/). Based on the transcriptomic data provided by Wang et al. (2017), the transcript levels of these *Sjp_npps* were determined (https://parasite.wormbase.org/expression/schistosoma_japonicum_prjea34885/index.html#SRP090154). On the left side, transcript profiles of the 10 *Sjp_npps* in females are presented, on the right side those of males. A reduction of *Sj_npp* transcript levels was observed in females after pairing from d18 on. In contrast, the transcript levels of these *Sj_npps* remained at a constant level in males or, in one case (Sjp_0097680, potential ortholog of Smp_052880), increased from d18 on.

Wang, J., Yu, Y., Shen, H., Qing, T., Zheng, Y., Li, Q., et al. (2017) Dynamic transcriptomes identify biogenic amines and insect-like hormonal regulation for mediating reproduction in *Schistosoma japonicum*. *Nat. Commun.* 8:14693. doi: 10.1038/ncomms14693.
